# The long-term ingestion of a diet high in extra virgin olive oil produces obesity and insulin resistance but protects endothelial function in rats: a preliminary study

**DOI:** 10.1186/1758-5996-5-53

**Published:** 2013-09-18

**Authors:** Hady Keita, Eduardo Ramírez-San Juan, Norma Paniagua-Castro, Leticia Garduño-Siciliano, Lucía Quevedo

**Affiliations:** 1 Departamento de Fisiología, Escuela Nacional de Ciencias Biológicas, Instituto Politécnico Nacional, Carpio y Plan de Ayala, México, D.F., México; 2 Farmacia de la Escuela Nacional de Ciencias Biológicas, Instituto Politécnico Nacional, México, D.F., Mexico

**Keywords:** Endothelial dysfunction, Obesity, Extra virgin olive oil, Insulin resistance

## Abstract

**Background:**

It has been hypothesized that fatty acids derived from a diet high in saturated fat may negatively affect endothelial function more significantly than a diet high in unsaturated fat; nevertheless, the effects of the long-term ingestion of monounsaturated fatty acids on endothelial function have been poorly studied.

**Methods:**

To examine the chronic effects of monounsaturated (e.g.*,* extra virgin olive oil (EVOO)) or saturated (e.g.*,* margarine (M)) fatty acid-rich diets on the development of insulin resistance and endothelial dysfunction in rats, three groups of rats were fed control, high-EVOO or high-M diets for 20 weeks. Body weight, energy consumption, insulin resistance, lipid peroxidation and *in vitro* vascular reactivity with and without metformin were assessed during the study period.

**Results:**

Both high-fat diets produced obesity and insulin resistance. EVOO-fed rats showed smaller increases in total cholesterol and arterial lipid peroxidation when compared with M-fed rats. Vascular reactivity to phenylephrine and sodium nitroprusside was not modified, but the vasodilating effect of carbachol was especially reduced in the M-fed rats compared with the EVOO-fed or control groups. Metformin addition to the incubation media decreased the vascular response to phenylephrine; decrease that was lower in rats fed with both high fat diets, and increased the carbachol and nitroprusside effects, but the metformin-enhanced response to carbachol was lower in the M group.

**Conclusions:**

Our results suggest that feeding rats with high quantities of EVOO, despite producing obesity and insulin resistance, produces low levels of circulating cholesterol and arterial lipoperoxidation compared to M fed rats and shows a preserved endothelial response to carbachol, effect that is significantly enhanced by metformin only in rats fed with control and EVOO diets.

## Background

During the last few decades, sedentary lifestyles and transient changes in consumption patterns, including a sharp increase in fat consumption from vegetable oils and animal-based food sources, have led to drastic increases in the prevalence of overweight and obese individuals in many developed and developing countries [1]. The importance of fat consumption in the development of insulin resistance, type 2 diabetes mellitus and cardiovascular disease has also been shown [2,3], but differences in weight gain and predisposition to cardiovascular disease in some populations suggest that dietary fat composition plays an important role in determining the development of these chronic diseases [4,5]. Diets rich in saturated fatty acids (SAFA) have been linked to endothelial dysfunction, which contributes to macrovascular alterations. On the other hand, the Mediterranean diet, which is mainly characterized by abundant vegetables, fruits, complex carbohydrates, large amounts of olive oil as the main dietary fat, low fat dairy and animal products which guarantees the low consumption of saturated fats, has been recognized for its positive effects on cardiovascular health [6,7] and its protective effect against the development of obesity and diabetes [8-10]. In the Mediterranean region, total daily lipid intake may contribute with the 40% of total energy intake in Greece, or 30% in Italy, hence, olive oil plays a central role in this diet [11]. Many studies have attributed some of the beneficial effects of the Mediterranean diet to olive oil phenolic compounds with high antioxidant capacity, e.g., hydroxytyrosol, tyrosol, oleuropein aglycon and its derivatives, which are naturally found in extra virgin olive oil and to the high proportion of monounsaturated fatty acids (MUFA), namely oleic acid [12-14]. EVOO polyphenols have been reported having strong anti-inflammatory properties [14], decrease oxidized low-density lipoproteins, decrease blood pressure and improve endothelial function in humans [15] and prevent oxidative DNA damage [16]. Interestingly, in a recent study, an extra virgin olive oil, rich in oleic acid, has been shown having antioxidant properties and an improved adaptive response of the body in rats submitted to exhaustive exercise [17].

It has also been reported that MUFA consumption leads to a decrease in low-density lipoprotein (LDL) cholesterol [18], while saturated fat intake increases LDL levels [19,20]. Concurrently, other studies have suggested that unsaturated fat increases LDL cholesterol in a manner similar to what has been observed with saturated fat, but saturated fat, especially trans fats, simultaneously decreases high-density lipoprotein (HDL) cholesterol levels and increase the deposition of cholesterol into cellular plasma membranes in vascular tissues, leading to the development of atherosclerosis over time [21,22]. The general consensus is that the dietary intake of SAFA increases cardiovascular risk but that MUFA and polyunsaturated fatty acids (PUFA) intake decreases risk [23]. Contrarily, it has been shown that increased circulating concentrations of cholesterol and free fatty acids reduce the endothelial bioavailability of nitric oxide (NO) due to both the increased generation of superoxide and a reduction in endothelial NO synthase activity [24,25]. Reactive oxygen species can react with the cell membrane PUFAs, causing lipid peroxidation and thus altering the vasodilatory function of the endothelium [26]. Furthermore, adipose tissue secretes free fatty acids and cytokines, a process that is intimately involved in the development of insulin resistance and contributes to a proinflammatory state which, overall, puts a patient at an increased cardiovascular risk [27-29]. Many studies have shown that vascular complications are a major cause of mortality in diabetic patients [30] and that the endothelium plays a critical role in controlling vascular tone and blood flow by synthesizing mediators of vasodilation and vasoconstriction.

Impaired endothelium-dependent vasodilation in obesity-associated insulin resistance [31] and diabetes [32] is characterized by a diminished NO production and the development of a pro-inflammatory vascular phenotype that promotes atherosclerosis and adverse cardiovascular events. Previous studies of rats fed with high-fat diets enriched with olive oil for 4 weeks showed that the rats achieved low body weights [33] and retained a normal insulin sensitivity [34]. In contrast, Buettner et al. [35] found that feeding rats a diet high in EVOO over a long period of time produces obesity and insulin resistance, similar to what is seen in animals fed a lard-based diet. These findings suggest that obesity and the deleterious effects on vascular integrity of ingesting high quantities of fats may only occur with chronic ingestion. Most of the recent studies on beneficial effects of EVOO were conducted with either well balanced or hypocaloric diets, but there are scant and contradictory data about the effects due to a high EVOO ingestion, therefore the aim of this study was to determine if the long-term consumption of a hypercaloric diet high in monounsaturated fatty acids such as the extra virgin olive oil produces obesity, insulin resistance and endothelial dysfunction in rats as well as compare these effects with those of a hypercaloric diet high in saturated fat.

## Methods

### Animals and diets

Twenty seven male Wistar rats, obtained from the University of Hidalgo and initially weighing between 120–150 g (5–6 wk old), were housed in individual cages in a temperature-regulated room (22°C ± 2°C) with 12-hour dark–light cycles (lights on from 7:00 AM to 7:00 PM). After one week of acclimatization, nine rats were assigned to each diet group (control, EVOO, M), and the specialized feedings were initiated. The animals had free access to their respective diets. The control animals were fed a commercial diet (Rodent Laboratory Diet 5001 PMI, Richmond, IN), with a total energy content of 13.68 kJ/g. The remaining animals received a high-fat diet (25% extra virgin olive oil or margarine was added to the standard diet). Both high-fat diets had an energy content of 19.68 kJ/g (54.2% of total energy derived from fat). We used an Italian EVOO containing 474.2 mg/kg polyphenols that was purchased in a local market. The total phenol content was determined colorimetrically at 725 nm following the Folin & Ciocalteau method in the extracts in triplicate as previously described [36] and expressed as milligrams of galic acid equivalents per kilogram of oil. The EVOO comprised 15.44% saturated fat, 74.47% unsaturated fat and 10.07% polyunsaturated fat; while, the M comprised 57.14% saturated fat, 25.71% unsaturated fat and 17.14% polyunsaturated. The macronutrient compositions of the diets are shown in Table 1. The food consumption per animal was measured daily, and body weights were determined for each animal once a week over the 20-week period. All of the experimental procedures were conducted according to the recommendations of the *Guide for the Care and Use of Laboratory Animals* of the Mexican Council for Animal Care (NOM-062-ZOO-1999) and were approved by the National School of Biological Sciences Ethics and Biosecurity Committee.

**Table 1:** Macronutrient composition of study diets (g/100 g)

**Component g/100 g**	**Control diet**	**EVOO**	**Margarine**
		**High-fat diet**	**High-fat diet**
**Protein**	23.0	17.25	17.25
**Carbohydrate**	48.7	36.53	36.53
**Total fat**	4.5	28.37	28.37
**Fat from chow**	4.5	3.37	3.37
**EVOO**	-	25.0	-
**Margarine**	-	-	25.0
**Fiber**	6.0	4.5	4.5
**Minerals**	7.0	5.3	5.3
**Vitamins**	1.0	0.75	0.75
**Moisture**	10.0	7.5	7.5

*EVOO* extra virgin olive oil.

### Reagents

(R)-(−)-phenylephrine hydrochloride, isoproterenol hydrochloride, sodium nitroprusside and metformin (1.1 dimethylbiguanide hydrochloride) were all purchased from Sigma and Sigma-Aldrich, and carbachol was purchased from Research Biochemicals International. Rat/mouse insulin ELISA kits were purchased from Millipore (Linco Research Inc., St. Charles, MO, USA).

### Blood and serum measurements

After twenty weeks of experimental feeding, blood samples were obtained from the tips of the rats’ tails at 8:00 AM, following an overnight 12-hour fast. The glucose levels were determined in whole blood with a glucose meter (Optium medisense, Abbott Laboratories, Oxford, United Kingdom). The samples (0.5 mL) were collected at the conclusion of the experiment to determine the total cholesterol, HDL-cholesterol and triglyceride (TG) levels using specific enzymatic-colorimetric kits (Randox, Crumlin, UK). The samples were centrifuged at 3500 rpm for 15 min at 4°C. Plasma LDL- cholesterol levels were calculated using the formula of Friedeward et al. [37].

### Blood glucose and serum insulin levels during the intraperitoneal glucose tolerance test

An intraperitoneal (IP) glucose tolerance test (GTT) was performed in the rats after an overnight fast of 12 hours. Basal blood samples were obtained from the tip of the tail, and a 35% glucose solution was intraperitoneally injected (3 g/kg body weight). At 15, 30, 60, 120 and 180 minutes post-glucose administration, blood glucose levels were determined [38]. Fasting blood samples (0.25 mL) were obtained in basal conditions and were centrifuged at 3500 rpm for 15 minutes at 4°C. The resulting serum was kept frozen at −70°C until insulin levels were determined [39]. Insulin resistance was evaluated using the homeostasis model assessment of insulin resistance (HOMA-IR), which is calculated as the product of the fasting serum insulin (μU/mL) and the fasting blood glucose (mg/dL) divided by 2430 [40].

### Intraabdominal fat pad weights

At week 20, the rats were anesthetized with diethyl ether and euthanized by decapitation. The thoracic region was immediately dissected, and a segment of aorta, between the arch and the diaphragm, was removed for use in the study of endothelial function. The abdominal region was then dissected, and the intraabdominal fat deposits were excised and weighed. Accurately weighed samples from the aorta, liver and heart were also taken for the lipoperoxidation assay.

### Lipid peroxidation assay

Lipid peroxidation in the artery, heart and liver was assessed by measuring the malondialdehyde (MDA) content, as described by Buege and Aust [41], but with minor modifications. Briefly, 50 mg of tissue was homogenized in 0.5 mL of cold phosphate buffer (pH 7.4), followed immediately with the addition of 1 mL of a reagent containing trichloroacetic acid, thiobarbituric acid and HCl [15% (w/v), 0.375% (w/v) and 0.25 N, respectively] to the homogenate. This solution was then heated for 60 min in a boiling water bath. After cooling, the flocculent precipitate was removed by centrifugation at 1000 g for 10 min. A spectrophotometer was used to determine the absorbance at 532 nm. A sample containing reagent, only, was used as a blank. The MDA content was calculated using an extinction coefficient of 1.56 × 10^-5^ M^-1^ cm^-1^. The protein content was determined using the Coomassie blue method [42], with bovine serum albumin as the standard.

### Measurement of isometric force

The segment of thoracic aorta was placed in oxygenated, modified Krebs-Hanseleit solution (KHS) (NaCl, KCl, NaHCO_3_, CaCl_2_, NaHPO_4_, MgSO_4_, dextrose and EDTA). The thoracic aorta was cleaned of loosely adhering fat and connective tissue. It was cut into 3-mm-wide rings and then placed in an oxygenated (95% O_2_, 5% CO_2_) bath with 10 ml of KHS, with one end of the specimen connected to a tissue holder and the other to a transducer (TSD125C-50 g, BIOPAC Systems Inc. Santa Barbara California, Model MP100). First, the aortic ring was equilibrated for 45 min under a resting tension of 2.0 g. After equilibration, the aortic rings were precontracted with phenylephrine (1 × 10^-6^ M), and after reaching a stable plateau phase were exposed to carbachol (1 × 10^-6^ M) to verify the functionality of endothelium. Contraction was evoked again with phenylephrine (1 × 10^-6^ M), then cumulative concentration-response curves to sodium nitroprusside or carbachol were determined from 1 × 10^-8^ to 1 × 10^-4^ M for the relaxation studies. In another set of experiments, cumulative concentration-response curves to phenylephrine (1 × 10^-8^ to 1 × 10^-4^ M) were generated.

Metformin has been widely used to increase glucose and lipid metabolism in patients and rodent models of type 2 diabetes mellitus and insulin resistance to improve glycemic control and lower blood pressure. However, the insulin resistance mechanisms to increase incidence of vascular disease are not completely understood [43]. Additionally, as metformin has been reported to relax isolated arterial preparations through a nitric oxide dependent mechanism [43], we hypothesized that addition of metformin to the incubation media should improve the response to vascular active substances, showing differential effects in relation to fat diet composition. Thus, after performing the previous experiments, the aortic ring segments were washed sufficiently to recover their basal contraction (2.0 g) and were incubated in metformin (10^-4^ M) for 40 min, finally, the first experiment was repeated using the same incubation medium.

### Statistical analyses

The results are presented as the means ± SEM, and one-way ANOVA, two-way RM-ANOVA or three-way RM-ANOVA analyses were used to compare the mean values among the different groups, followed by further analysis with the Student-Newman–Keuls’ *post hoc* test. In all cases, a P value of 0.05 or less (two-tailed) was considered statistically significant.

## Results

### Food consumption, energy intake and body weight

**Figure 1:**
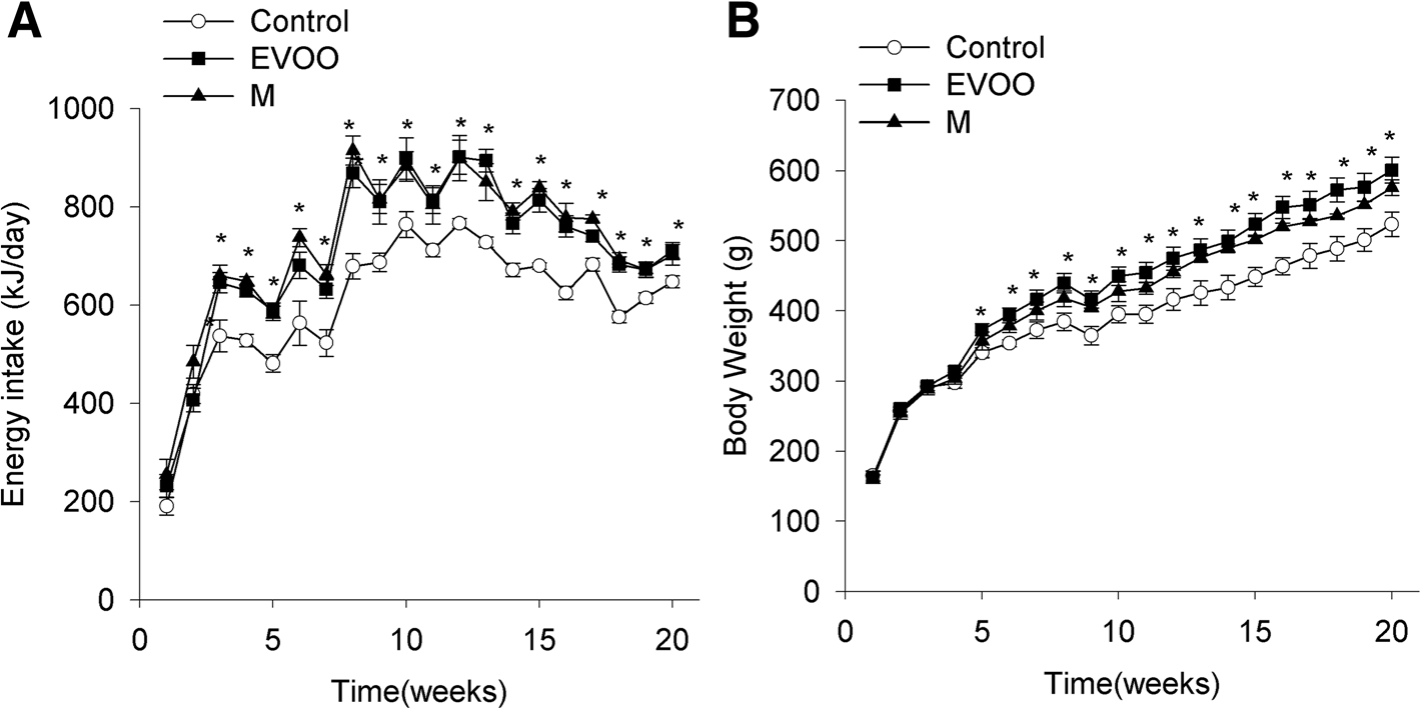
**Changes in body weight and energy intake.** Energy intake **(A)** and body weight **(B)** of rats fed with control or high-fat olive oil or margarine diets for 20 weeks. * P < 0.05, significant difference compared with the control group by 2-way RM ANOVA (n = 9 per group).

The rats fed with extra virgin olive oil and margarine diets ingested significantly less food than the control diet rats P < 0.05 (data not shown); however, the overall energy intake and body weights were significantly higher in both of the high-fat diet group rats (EVOO and M) than in the control group (P < 0.05; Figures 1A and 1B) at the conclusion of the 20 weeks of dietary treatment. No significant differences in these variables between the two high-fat diets were found.

### Glycemia and glucose tolerance

**Figure 2:**
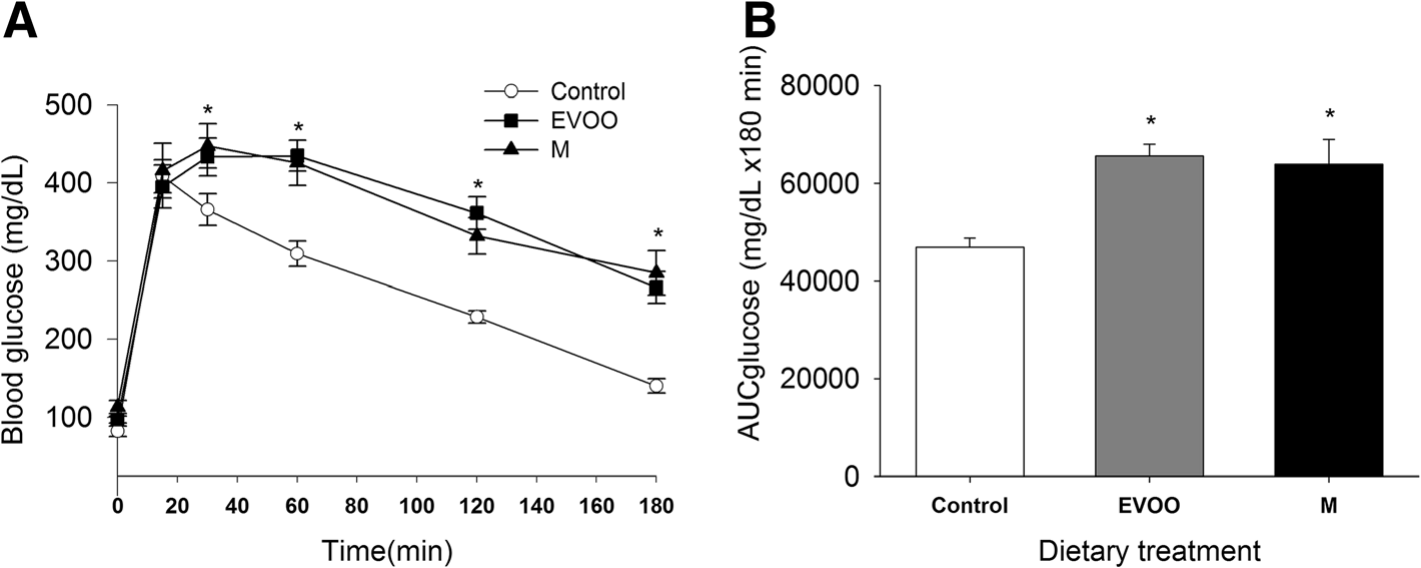
**Effect of the HF-diets on blood glucose levels.** Glycemia **(A)** and the area under the glucose curve **(B)** after 12 hours of fasting following an IP injection of 3 g/kg of glucose solution in rats fed a control or a high-fat diet (EVOO or M) for 20 weeks by 2-way RM ANOVA **(**for **A)** and one-way ANOVA **(**for **B)**. *P < 0.05, significant difference compared with the control group. (n = 9 per group).

At week 20 of dietary treatment, an IP injection of a glucose solution (3 g/kg) produced a significant increase in glycemia after 15 minutes in all animals. At 30 minutes, blood glucose levels decreased only in the control rats and were significantly lower in these rats compared with the HF-fed rats. At 180 minutes, the control diet group had recovered to their baseline blood glucose levels, but the HF-diet groups had not (Figure 2A). The area under glucose curve was significantly greater in the HF-diet groups than in the control group (Figure 2B). However, the corresponding insulin levels under fasting conditions were not different between the groups. The HOMA-IR analysis revealed significant differences in insulin resistance between the HF-diet groups and the control group (Table 2).

**Table 2:** Fasting blood glucose, plasma insulin levels and HOMA-IR after 20 weeks of dietary treatment

	**Control**	**EVOO**	**M**
**Glucose (mg/dL)**	92.5 ± 3.2	97.1 ± 4.2	113.1 ± 8.3*^,^&
**Insulin (μU/mL)**	22.51 ± 0.11	22.76 ± 0.52	22.93 ± 0.28
**HOMA-IR**	0.81 ± 0.03	1.07 ± 0.08*	1.14 ± 0.11*

Values are expressed as the means ± SEM (n = 9 per group). *P < 0.05, significant difference compared with the control group; & P <0.05, significant difference compared with the EVOO group by one-way ANOVA.

### Serum total cholesterol, lipoprotein cholesterol (HDL and LDL) and triglycerides (TG)

Total cholesterol, HDL, LDL and TG plasma concentrations after 20 weeks of dietary treatment varied between the experimental groups. M-fed rats showed higher total and LDL-cholesterol plasma levels than those found in the control or EVOO-fed rats (P < 0.05), but HDL and TG levels did not differ significantly between the dietary groups (Table 3).

**Table 3:** Serum total cholesterol, HDL, LDL and TG concentrations, and intraabdominal fat

	**Control**	**EVOO**	**M**
**Total Cholesterol (mg/dL)**	71.54 ± 4.6	72.31 ± 2.6	84.0 ± 5.0*
**TG (mg/dL)**	58.46 ± 5.3	67.32 ± 3.5	65.54 ± 4.4
**HDL cholesterol (mg/dL)**	19.72 ± 2.3	21.6 ± 2.3	18.9 ± 2.3
**LDL cholesterol (mg/dL)**	40.0 ± 5.2	37.3 ± 3.4	52.0 ± 5.0*
**Intraabdominal fat (g/100 g body weight)**	5.18 ± 0.57	9.06 ± 0.44*	8.71 ± 0.63*

* P < 0.05, significant difference compared with the control group by one-way ANOVA (n = 9 per group). Values are expressed as the means ± SEM.

### Intraabdominal fat pad weights

The combined weight of the retroperitoneal, epididymal and mesenteric fat pads, expressed as a percentage of body weight, was greater for the HF-diets rats than for the control rats (Table 3).

### Lipid peroxidation

Malondialdehyde levels in the thoracic aortas, hearts and livers of the M-diet group were significantly higher than the levels found in either the control or EVOO group (P < 0.05; Table 4).

**Table 4:** Malondialdehyde levels in the aorta, heart and liver

**Tissues**	**Control**	**EVOO**	**M**
**Arterial MDA (mM/mg Protein)**	15.76 ± 1.83	22.94 ± 4.58	50.46 ± 5.56*^,^&
**Heart MDA (mM/mg Protein)**	3.77 ± 0.34	3.83 ± 0.59	7.13 ± 0.20*^,^&
**Hepatic MDA (mM/mg Protein)**	3.09 ± 0.22	3.25 ± 0.34	5.62 ± 0.47*^,^&

Values are expressed as the means ± SEM (n = 9 per group). *MDA* malondialdehyde. * P < 0.05, significant difference compared with the control group. & P < 0.05, significant difference between the EVOO and M groups according to one-way ANOVA.

### *In vitro* aortic response to phenylephrine in the absence and presence of metformin

**Figure 3:**
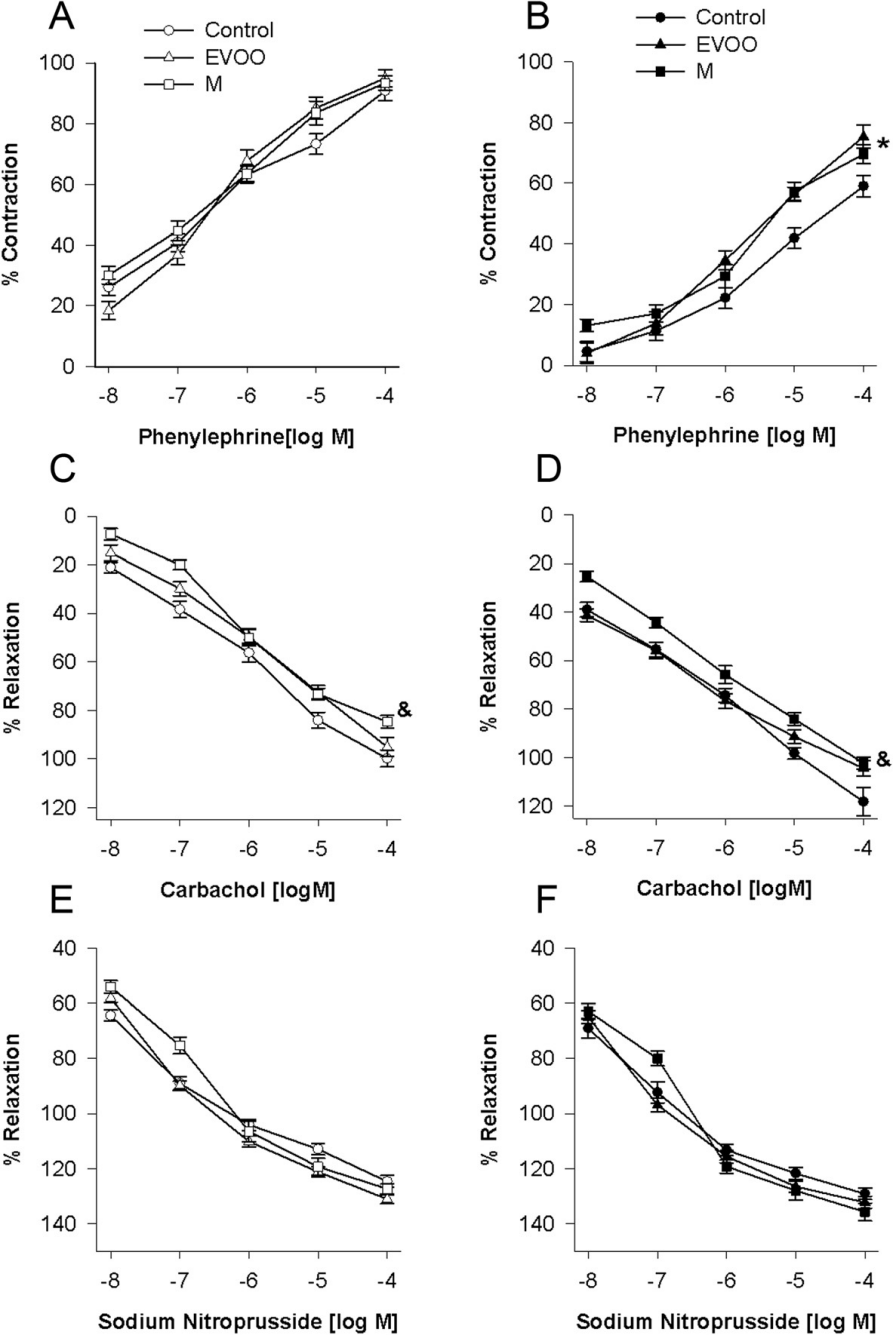
**Effects of the HF-diets on endothelial function.** Concentration-response curves for phenylephrine **(A)** and **(B)**, carbachol **(C** and **D)** or sodium nitroprusside **(E** and **F)** before and after metformin addition to the incubation media of thoracic aortic rings obtained from age-matched control, EVOO and M rats (n = 9 per group). The results show the contraction of the thoracic aortic rings as a percentage of the maximal contraction induced by phenylephrine (10 ^-8^ to 10^-4^ M) and the relaxation as a percentage of the effect induced by carbachol (1 × 10^-8^ to 1 × 10^-4^ M) or sodium nitroprusside (1 × 10^-8^ to 1 × 10^-4^ M). * P < 0.05 significant difference compared with the control group; & P < 0.05 significant difference compared with the control and the EVOO groups by 2-way RM ANOVA. Values are the means ± SEM.

In generating the concentration-response curves for phenylephrine, there were no significant differences detected between the diet groups (P > 0.05) (Figure 3A). However, incubation of the aortic rings with metformin (10^-4^ M) significantly reduced the vasoconstrictor response (P < 0.05) to phenylephrine, but the effect was lower in both high-fat diet groups than in the control group (Figure 3B).

### Relaxation response to carbachol or sodium nitroprusside in the absence or presence of metformin

The addition of phenylephrine (1 × 10^-6^ M) to the incubating medium induced a maximum contraction that reached a plateau. Subsequently, the concentration-response curve for sodium nitroprusside was determined for each group (1 × 10^-8^ to 1 × 10^-4^ M), both in the presence and absence of metformin. The relaxing effect of carbachol was significantly lower in the M-fed rats compared with the EVOO-fed or control groups (Figure 3C). Metformin increased the relaxation produced by carbachol in all of the dietary groups, but the metformin effect was less pronounced in the M-fed group (Figure 3D). There were no significant differences in the relaxation response to sodium nitroprusside of the experimental groups, either in the presence or absence of metformin (P > 0.05), but there was a significant difference within each group in their responses to sodium nitroprusside before and after the addition of metformin to the incubation medium (Figure 3E and 3F).

## Discussion

The high consumption of energy-dense vegetable fat and physical inactivity are considered major causes of obesity. Many studies have demonstrated that high-fat diets have contributed to the increased prevalence of insulin resistance [44-46] and to the eventual development of diabetes in genetically susceptible individuals and populations [31,47,48]. This study shows that two different vegetable fat have different metabolic and vascular effects in rats subjected to high-fat diets of isoenergetic value. Ingesting high levels of extra virgin olive oil produces obesity and insulin resistance in study rats, which is comparable to what is observed in margarine-fed rats, but only the margarine-heavy diet produced hypercholesterolemia and significant endothelial dysfunction. In agreement with other reports [4,49,50] the rats fed HF diets ingested more energy and gained more weight than the control rats did. Contrary to our results, some studies support an anti-obesogenic effect of EVOO [33,51], but other longer-term studies report weight gain with sustained EVOO intake [35]. This discrepancy could be partly explained by the duration of dietary treatment (4 weeks vs. 12 weeks) or the quantity of EVOO administered [34]. Indeed, our results support that obesity depends on the quantity of fat ingested, its composition and the duration of dietary treatment. This is because high-fat feeds in rats require at least 4 weeks of treatment to produce a significant increase in body weight, and both monounsaturated and saturated fatty acids favor adiposity. In recent years, the adherence to the Mediterranean diet has been associated with lower risk for cardiovascular disease [6]. This diet is characterized by the high consumption of fruits, vegetables, cereals, fish and virgin olive oil. This Mediterranean dietary pattern has a low energy density and a high fiber content that contributes to satiation which may explain the lower body weight, abdominal fat deposition and fasting blood glucose reported with the adherence to this diet [7,52]. In contrast, our results show that feeding rats with the hypercaloric and unbalanced high-fat diets based on EVOO or M, produce an increase in energy ingestion that may lead to the accumulation of visceral fat, which in turn is linked to the progression of insulin resistance and impaired glucose tolerance [35,53,54]. In our rats, 3 hrs after the administration of a glucose load, blood glucose levels had decreased to their basal levels in the control group, only. The lower capacity of rats maintained on HF diets (EVOO or M) to return to their basal levels of glucose is indicated by the greater areas under their glucose curves (Figure 2). Moreover, the HOMA-IR analysis, which indicates insulin resistance under fasting conditions, revealed significant differences between the HF-diet groups and the control group, with the HF-fed rats showed signs of insulin resistance. Consistent with this result, it has already been shown that HF diets contribute to glucose intolerance and the subsequent development of insulin hypersecretion after a glucose load [50,55] because the body attempts to keep glucose at normal physiological levels. Several studies have reported that visceral adipocytes are less responsive than subcutaneous fat cells to the antilipolytic effect of insulin [56,57] and that exposure of the liver to high concentrations of free fatty acids can induce changes in insulin signaling that promote hepatic insulin resistance [58]. In summary, the metabolic impact of high oleic acid intake on glucose and lipid metabolism is not conclusive in humans, whereas it clearly leads to obesity and insulin resistance in rats [35]. Although, dietary fat composition plays an important role in the prevention of cardiovascular disease (CVD) and metabolic syndrome [59,60], it has been suggested that the total intake of fat (SAFA, MUFA and PUFA), regardless of the quality of the fat, can increase the risk of CVD [23]. In this study, EVOO and M diets have shown to exert different effects on the vasomotor response of endothelial tissue, showing signs of altered function in the margarine-fed group, only. Extra virgin olive oil is rich in unsaturated fatty acids, mainly oleic acid, and phenolic compounds [12], which contribute to its cardioprotective effects [14]. In contrast to EVOO, margarine has a high saturated fatty acid content, which is produced industrially via the hydrogenation of unsaturated oils [61]. Many studies have shown that consumption of these fats contributes to an increased cardiovascular risk [23]. As our results show, lipid peroxidation was elevated only in the rats that were maintained on a high-M diet. These results suggest that the consumption of a high-M diet, over a period of 20 weeks, leads to increased levels of oxidative stress, hypercholesterolemia and insulin resistance which in turn lead to alterations in tissue function, including endothelial vasomotor response. These findings are consistent with many studies that have shown that the antioxidant properties of EVOO are mainly due to its high content of polyphenolic compounds [12], which are strong antioxidants and radical scavengers [62,63]. In contrast, saturated fatty acids increase oxidative stress and thus modify the lipid composition of cell membranes [64,65]. Consistent with these studies, our results indicate that the serum lipid profiles of rats maintained on the M diet reflected higher levels of total and LDL cholesterol compared with the rats fed high-EVOO or control diets. It is well documented that hypercholesterolemia is associated with a higher incidence of atherosclerosis [66]. In addition, although there was no significant difference in the levels of TG, there was an increasing trend in this parameter in M-fed rats, which suggests that, even though all of the rats that were fed high-fat diets (EVOO or M) were obese, the EVOO-high diet still conferred a lower cardiovascular risk compared with the M-high diet. Given that obesity has been linked to insulin resistance and increased inflammation, causing endothelial dysfunction, EVOO-fed rats should also be expected to show signs of altered endothelial function. Carbachol-induced NO production and vasodilation are conserved in these rats, however, suggesting that the expected endothelial damage had been prevented. It has been shown that oleic acid, the major fatty acid constituent in EVOO and polyphenols, diminishes the oxidation of olive oil-derived lipoproteins, thus mitigating any potential oxidative damage to endothelial vasomotor function. These results support the finding that, among EVOO compounds, polyphenols, mainly hydroxytyrosol and tyrosol, increase NO production and prevent the formation of the powerful oxidant peroxynitrite [26]. The concentration-response curve for phenylephrine showed that the high-fat diets did not modify the endothelium’s vasoconstrictive response to this substance, nor did they affect the tissue’s vasodilatory response to sodium nitroprusside. However, the M diet did reduce the relaxation generated by carbachol, suggesting that the ingestion of significant amounts of saturated fat affected the endothelium-dependent vasorelaxation response in the rats fed the M diet. Hypercholesterolemia and high levels of lipid peroxidation in the arteries are likely to be associated with the alteration in vascular response because it is well known that inflammation plays a key role in the onset of atherosclerosis and oxidative stress and may also contribute to increased endothelial damage [67]. Earlier studies support the hypothesis that endothelial dysfunction is associated with a reduction in the release [68] or availability of NO, the production of which is downregulated in the presence of reactive oxygen species (ROS), the production of which, in turn, is generally increased in the obese state [31]. Although the results of this study showed an increased aortic lipoperoxidation in M fed rats, and there were significant differences in endothelial response to vasorelaxing substances among HF diets groups, the differences in endothelial reactivity were lower than was expected for the high values of lipoperoxidation in M-fed rats, suggesting that the quantity of fats in the diets (25% w/w) was very high and duration of the regimen diet (20 weeks) was too long, producing some common alterations not related to lipoperoxidation in both HF rat diets. Furthermore, there were no high differences when comparing to control rats, thus indicating as previously suggested that the process of maturation and aging in rat lead to degenerative changes at the cellular level including arterial endothelial cells function [69]. Indeed, a reduction in the dietary intervention could show higher differences in endothelial function between EVOO and M diets. As our results show, there was no significant difference in the vasodilatory responses to sodium nitroprusside in the arteries from the experimental groups, which suggests that the limited vasodilatory response to carbachol (muscarinic agonist) that was observed in the arteries of the M-fed rats was due to a lower endothelial production of NO. The diets did not affect the endothelial response to alpha-1-adrenergic (phenylephrine) or beta-adrenergic (isoproterenol, results not shown) agonists, which may indicate that the diets had no effect on the adrenergic vascular response. Furthermore, this study corroborates previous findings that incubation of the aorta rings from streptozotocin-induced diabetic rats with metformin enhanced the carbachol-induced relaxation and reduced the phenylephrine-induced contraction, which have been reported to be endothelium dependent [70], similarly, in our insulin resistant rats, the addition of metformin to the incubation media showed a lower reduction of the phenylephrine-induced contraction in both high fat diet fed rats compared to control rats, but an even lower response to carbachol in M rats, indicating that just high M diet altered both endothelium dependent relaxing mechanisms. Metformin vasodilatory effects have been proposed to be associated with the activation of AMP-activated protein kinase which increases the activity of endothelial NO synthase [71]. Additionally, the enhanced vasodilator effect of nitroprusside indicates that metformin also produces relaxation through a mechanism that is independent of endothelial NO production, possibly via the decreased production of vasoconstricting prostanoids [72] or decreased intracellular calcium [73], both of which are factors that could be altered and by doing so, contributing to lower the endothelial response in M-fed rats.

## Conclusions

In conclusion, we found that the long-term high-fat intake lead to obesity and insulin resistance, regardless of the fat composition (saturated or monounsaturated). Nevertheless, EVOO did not increase plasma levels of total cholesterol or LDL cholesterol, nor did it increase arterial lipid peroxidation, which may contribute to the preservation of endothelial response to carbachol, effect that is significantly enhanced by metformin only in rats fed with control and EVOO diets. Further to this preliminary study, longer-term animal and *in vitro* studies are needed to better understand the mechanisms regarding the ingestion of a diet high in extra virgin olive oil to produce obesity and insulin resistance but protect endothelial function.

## Abbreviations

CVD: Cardiovascular disease; EVOO: Extra virgin olive oil; GTT: Glucose tolerance test; HDL cholesterol: High-density lipoprotein cholesterol; HF-diet: High-fat diet; HOMA-IR: Homeostatic model assessment of insulin resistance; IP: Intraperitoneal; KHS: Krebs Hanseleit solution; LDL cholesterol: Low-density lipoprotein cholesterol; M: Margarine; MDA: Malondialdehyde; MUFA: Monounsaturated fatty acids; PUFA: Polyunsaturated fatty acids; ROS: Reactive oxygen species; SAFA: Saturated fatty acids; TG: Triglycerides.

## Competing interests

The authors declare that they have no competing interests.

## Authors’ contributions

Conceived and designed the experiments: ERSJ, and LQ. Performed the experiments and analyzed the data: HK, ERSJ, LGC and NPC. HK and NPC participated in its design and coordination and helped to draft the manuscript. Wrote the paper: LQ. All authors read and approved the final manuscript.
